# Ectodermal Organ Development Is Regulated by a *microRNA-26b-Lef-1-Wnt* Signaling Axis

**DOI:** 10.3389/fphys.2020.00780

**Published:** 2020-07-14

**Authors:** Steve Eliason, Thad Sharp, Mason Sweat, Yan Y. Sweat, Brad A. Amendt

**Affiliations:** ^1^Department of Anatomy and Cell Biology, The University of Iowa, Iowa City, IA, United States; ^2^Craniofacial Anomalies Research Center, The University of Iowa, Iowa City, IA, United States; ^3^Iowa Institute for Oral Health Research, The University of Iowa, Iowa City, IA, United States

**Keywords:** *microRNA-26b*, *Lef-1*, Wnt signaling, ectodermal organ, stem cells, *miR-26b* mouse models

## Abstract

The developmental role of *Lef-1* in ectodermal organs has been characterized using *Lef-1* murine knockout models. We generated a *Lef-1* conditional over-expression (COEL) mouse to determine the role of *Lef-1* expression in epithelial structures at later stages of development after endogenous expression switches to the mesenchyme. *Lef-1* over expression (OE) in the oral epithelium creates a new dental epithelial stem cell niche that significantly increases incisor growth. These data indicate that *Lef-1* expression is switched off in the dental epithelial at early stages to maintain the stem cell niche and regulate incisor growth. Bioinformatics analyses indicated that *miR-26b* expression increased coinciding with decreased *Lef-1* expression in the dental epithelium. We generated a murine model over-expressing *miR-26b* that targets endogenous *Lef-1* expression and *Lef-1*-related developmental mechanisms. *miR-26b* OE mice have ectodermal organ defects including a lack of incisors, molars, and hair similar to the *Lef-1* null mice. *miR-26b* OE rescues the *Lef-1* OE phenotype demonstrating a critical genetic and developmental role for *miR-26b* in the temporal and spatial expression of *Lef-1* in epithelial tissues. *Lef-1* expression regulates Wnt signaling and Wnt target genes as well as cell proliferation mechanisms, while *miR-26b* OE reduced the levels of Wnt target gene expression. The extra stem cell compartment in the *COEL* mice expressed *Lef-1* suggesting that *Lef-1* is a stem cell factor, which was absent in the *miR-26b OE/COEL* rescue mice. This is the first demonstration of a microRNA OE mouse model that has ectodermal organ defects. These findings demonstrate that the levels of *Lef-1* are critical for development and establish a role for *miR-26b* in the regulation of ectodermal organ development through the control of *Lef-1* expression and an endogenous stem cell niche.

## Introduction

MicroRNAs (miRs) play an important role in the development of craniofacial structures. A conditional craniofacial specific knockdown of miRs lead to specific tooth defects and growth defects. An oral epithelial specific *Pitx2^*Cre*^* crossed to a *Dicer1 floxed* mouse demonstrated that teeth developed abnormally when *Dicer1* expression was ablated in the oral epithelium (Cao et al., 2010). One of the mature miRs expressed and deleted by *Pitx2^*Cre*^/Dicer1^*Flox/Flox*^* in the murine craniofacial region was *miR-26b* (Cao et al., 2010).

*miR-26b* expression is widespread in different tissues at different stages of development. Established roles have been determined for *miR-26b* in tissue/organ growth and several cancer models (Zhang et al., 2010; Gao et al., 2011) and we have reported that *miR-26b* can act as a tumor suppressor in a colon cancer model (Zhang et al., 2014). In the pituitary, *miR-26b* directly binds the *3′UTR* of the Lymphoid enhancer-binding factor 1 gene (*Lef-1) in vitro* and *in vivo* and regulation of *Lef-1* may promote Pit-1 lineage differentiation during pituitary development (Zhang et al., 2010). A direct role for *miR-26b* in ectodermal organ development has not been reported.

*Lef-1* plays a critical role in organ, craniofacial and tooth development. The Lef-1 protein shares homology with HMG family proteins and has been shown to act as a transcription factor (Travis et al., 1991). *Lef-1* is required for the development of multiple organ systems, including hair and tooth development and its role in Wnt signaling has been established (Travis et al., 1991; Van Genderen et al., 1994). In the developing tooth bud, *Lef-1* is expressed in the oral and dental epithelium at embryonic day E10.5, followed by a transition to mostly mesenchymal expression in the developing tooth bud starting at E14.5 (Kratochwil et al., 1996; Sasaki et al., 2005; Sun et al., 2016). The *Lef-1* general knockout has tooth developmental defects and a complete arrest of molar and incisor development at the late bud stage, but earlier stages of tooth development appear normal (Van Genderen et al., 1994). In addition, growth defects, abnormal hair/fur, and a kink in the tail have been described in the *Lef-1* null mice. *Lef-1* has been shown to be important for the regulation of *Fgf4* expression and over-expression of *FGF4* can rescue the late stage tooth bud delay in *Lef-1^–/–^* mice and FGF activation is thought to induce interactions between the dental epithelium and the dental mesenchyme (Kratochwil et al., 1996). Interestingly, in the murine epidermis and hair follicles, *Lef-1* establishes stem and progenitor cell compartments (Reya and Clevers, 2005; Huang and Qin, 2010; Petersson et al., 2011).

The transcription factor *Sox2* is required for the development of several endodermal tissues, including the trachea (Xie et al., 2014), stomach and gut (Que et al., 2007), and ectodermal tissues including the anterior pituitary (Jayakody et al., 2012), lens epithelium (Taranova et al., 2006), tongue epithelium (Balaguer et al., 2011) and hair follicles (Clavel et al., 2012). We have recently shown that conditional inactivation of *Sox2* leads to lower incisor arrest at E16.5 and abnormal dental development due to decreased stem cell proliferation (Sun et al., 2016). *Sox2* and *Lef-1* epithelial expression domains are juxtaposed in the murine oral epithelium and dental placode (Sun et al., 2016). Interestingly, ablation of either *Sox2* or *Lef-1* results in arrested tooth development at early developmental stages (Van Genderen et al., 1994; Kratochwil et al., 1996; Sun et al., 2016).

To define the role of *Lef-1* during embryonic development we used a conditional over-expression (COEL) of *Lef-1* (*COEL*) murine model to determine if dental epithelial specific over-expression would affect craniofacial and dental development (Sun et al., 2016). The ectopic over-expression of *Lef-1* in the epithelium leads to alterations in the labial cervical loop (LaCL) morphology and enhanced incisor growth, altered regulation of *Sox2*, enhanced proliferation, altered *Amelogenin* expression and the regulation of Wnt associated gene expression. In addition, we created a *EF1a* promoter-*miR-26b* transgenic over-expression mouse (*miR-26b* OE) to establish the role of *miR-26b* in craniofacial and tooth development. The *miR-26b OE* mouse shows growth defects, abnormal hair/fur, a crook in the tail and a complete lack of molar or incisor development, all phenotypes that are shared by the *Lef-1* knockout mouse (Van Genderen et al., 1994). Furthermore, we mated the *miR-26b OE* mice with the *COEL* mice and were able to rescue the *COEL* incisor growth phenotype. These studies demonstrate that *Lef-1* expression levels are critical for incisor growth. The correct *Lef-1* dosage is required because ablation of epithelial *Lef-1* causes tooth development to arrest at E14.5 and that an increase in epithelial *Lef-1* results in an expanded dental epithelial stem cell (DESC) niche and over-grown incisors. These finding correlate with increased levels of *miR-26b* in the dental epithelium after E14.5 at which stage *Lef-1* is normally decreased to allow for normal tooth development. Thus, our research demonstrates that; (1) precise *Lef-1* levels are critical for tooth development; (2) *miR-26b* is an important regulator of *Lef-1* in tooth development; (3) *Lef-1/miR-26b* work together to regulate the Wnt response required for normal tooth development, growth and maintenance; and (4) *miR-26b* expression in the dental stem cell niche after E14.5 reduces *Lef-1* expression required to maintain the stem cell niche and normal incisor growth.

## Materials and Methods

### Mouse Lines and Embryonic Staging

Mice were housed and experiments performed according to the Office of Animal Resources guidelines at the University of Iowa. The *Lef-1 COEL* mouse line was generated by inserting *Lef-1* downstream of a CAAG promoter and a floxed transcription stop signal (Sun et al., 2016). The *miR-26b* transgenic mouse was derived by insertion of an *EF1a* promoter-*miR-26b-5p* (61 bp) construct into mice by pronuclear injection. After backcrossing to C57BL/6 mice multiple times, the location of the insertion was determined by genomic sequencing. The *Pitx2*^*Cre*^ mouse used in this study has been described previously (Liu et al., 2003). Each mouse line derived was crossed to a C57BL/6 background. For embryonic staging experiments, the observed vaginal plug date of the female was designated as E0.5. Embryos were collected on the required date, and genomic DNA was isolated from a portion of the embryonic or neonatal material (usually the tail) for genotyping. The genotyping primers for all the mouse lines are listed in Table 1.

**TABLE 1:** A list of the primers used for genotyping and qPCR.

**Genotype primers:**
Cre: GCATTACCGGTCGATGCAACGAGTGATG GAGTGAACGAACCTGGTCGAAA TCAGTGC
Lef-1 cKI: TGAGGCGGAAGTTCCTATTCT GGCGGATCACAAGCAATAAT
Lef-1 WT: TCCCAAAGTCGCTCTGAGTT GGCGGATCACAAGCAATAAT
Mir26bTg+: TCAAGCCTCAGACAGTGGTTC AGTAATGGAGAACAGGCTGG
**RT primers**
Lef-1: TCACTGTCAGGCGACACTTC ATGAGGTCTTTTGGGCTCCT
Sox2: ATGCACAACTCGGAGATCAG TGAGCGTCTTGGTTTTCCG
Pitx2: CTGGAAGCCACTTTCCAGAG AAGCCATTCTTGCACAGCTC
Actb: GCCTTCCTTCTTGGGTATG ACCACCAGACAGCACTGTG
Axin2: ATGAGTAGCGCCGTGTTAGTG GGGCATAGGTTTGGTGGACT
Fgf7: TGGGCACTATATCTCTAGCTTGC GGGTGCGACAGAACAGTCT
Wnt5a: CAACTGGCAGGACTTTCTCAA CCTTCTCCAATGTACTGCATGTG
Tcf7: ACGAGCTGATCCCCTTCCA CAGGGACGACTTGACCTCAT
Lef1: GCCACCGATGAGATGATCCC TTGATGTCGGCTAAGTCGCC
Bmp4: ATTCCTGGTAACCGAATGCTG CCGGTCTCAGGTATCAAACTAGC
Nanog: CACAGTTTGCCTAGTTCTGAGG GCAAGAATAGTTCTCGGGATGAA
Pou5f1: AGAGGATCACCTTGGGGTACA CGAAGCGACAGATGGTGGTC
Mmp2: TGTCTTGCGTCTGACACTGC CTCCTTTGGGCTAGGTATCTCT
Fgf8: AGAGCCTGGTGACGGATCA CTTCCAAAAGTATCGGTCTCCAC
Ccnd2: GAGTGGGAACTGGTAGTGTTG CGCACAGAGCGATGAAGGT
miR-26b: TTCAAGTAATTCAGGATAGGTT Qiagen Univ rev

### Plasmid Constructs and Reporter Assays

*Lef-1* promoter luciferase and *pSil-miR-26b* constructs were previously described (Amen et al., 2007; Zhang et al., 2014). We used our recently described plasmid-based microRNA inhibitor system (PMIS) to generate a specific *PMIS-miR-26b* inhibitor (Cao et al., 2016). The luciferase TOP flash and FOP flash reporter constructs were purchased from EMD/Millipore (Burlington, MA, United States). Luciferase assays were done as previously described (Sun et al., 2016).

### Immunohistochemistry, Immunofluorescence, and Histology

Mouse embryos and tissue morphology was examined by Hematoxylin and Eosin staining procedure as done previously (Sun et al., 2016). Primary antibodies against Lef-1 (Cell signaling, Danvers, MA, United States), Myc (Santa Cruz, Dallas, TX, United States), Ki67 (Abcam, Cambridge, MA, United States), Sox2 (Abcam, Cambridge, MA, United States), and Amelogenin (Santa Cruz, Dallas, TX, United States) were then added to the sections. Incubation with primary antibody occurred overnight at 4°C. The slides were treated with FITC (Alexa-488)- or Texas Red (Alexa-555)-conjugated Secondary antibody and then were incubated for 30 min at room temperature for detection (Invitrogen, Carlsbad, CA, United States). Nuclear counterstaining was performed using DAPI-containing mounting solution. Pictures were taken under confocal microscope Zeiss 700 and photo preparation done on adobe photoshop. For some sections, photos were quantitated for fluorescence intensity using ImageJ.

### Imaging and Microcomputed Tomography (μCT)

Mouse skulls from three experimental and control animals were scanned with a Siemens Inveon Micro-CT/PET scanner using 60 kVp and 500 mA with a voxel size of 30 μm. Reconstructed images were imported using Osirx DICOM software. Mouse heads were prepared by overnight fixation at 4°C, followed by storage in 70% EtOH. Scans directed across the anterior-posterior plane produced 2D images which were matched between animals using topology markers such as the molar.

### RNA Isolation and Quantitative Real-Time PCR Gene Expression Analysis

RNA was isolated from dissected mouse tissues (mouse mandibles or dissected dental epithelium and mesenchyme tissues from the tooth germ) using RNA easy and miRNA easy kits (Qiagen, Carlsbad, CA, United States) and validated on agarose gels for purity, and qualitative assessment using rRNA bands to ensure minimal degradation. Nanodrop analysis gave precise concentration and cDNAs were generated by a mix of oligo DT and random hexamers and RT polymerase. cDNA quality was accessed by qPCR analysis to ensure consistency and melt curves and sequencing of qPCR products ensured the specificity of our probes. Experiments were done in triplicate and independent cDNAs were used to perform qPCR. ΔΔCT values were calculated and used to determine fold changes. qPCR primers are listed in Table 1.

### Wnt Array

Tissue was dissected from the mandibular region of a P0/P1 mouse and RNA was isolated as described (Sun et al., 2016). Using a Primer PCR Wnt signaling array (Bio-Rad, Hercules, CA, United States) cDNA from a WT or *miR-26b* overexpression mouse were compared according to the manufacture recommendations. RT primer probes were generated against genes that showed a twofold increase or decrease in the Biorad Wnt array plate and independently verified by qPCR.

### Statistical Analysis

For each condition, a minimum of three experiments was performed and error bars were presented as the ±SEM. An independent two-tailed *t*-test was used to determine the significance of differences between *WT*, *COEL*, *miR-26b*, and CO*EL/miR-26b* groups.

## Results

### Conditional Overexpression of *Lef-1* in the Oral and Dental Epithelium Results in Formation of an Extra Stem Cell Niche

**FIGURE 1:**
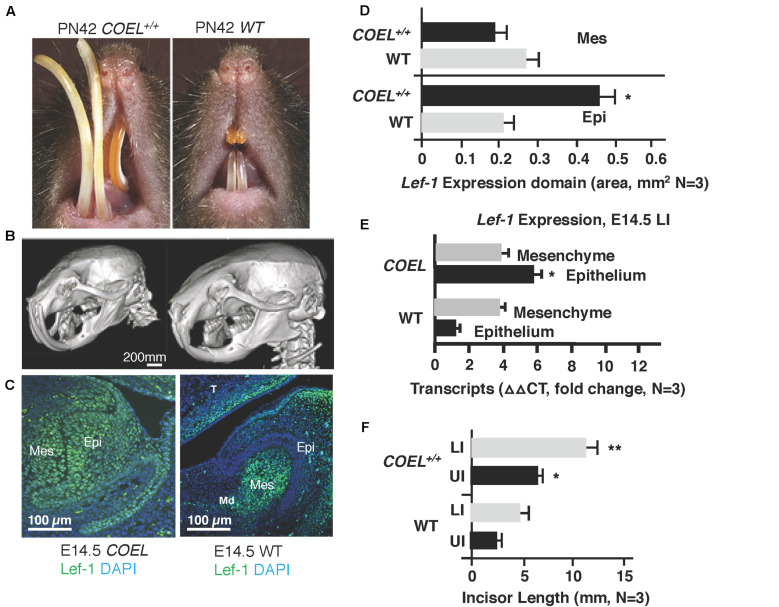
*Lef-1* conditional over-expression in the dental epithelium increases murine incisor growth. **(A)** The conditional over-expression of *Lef-1* (*COEL*) 42-day old (PN42) mice have an incisor overgrowth phenotype compared to WT mice. **(B)** μCT images to PN42 COEL mice show overgrown incisors and cranial bone defects. **(C)** Lef-1 expression by immunofluorescence shows Lef-1 ectopically expressed in the E14.5 dental epithelium of the COEL vs. WT (mesenchyme, Mes; epithelium, Epi). **(D)** Quantitation of the E14.5 *Lef-1* expression domains (mesenchyme, Mes; epithelium, Epi) from **(C)**. Three images, including the image in **(C)** were used to calculate expression domains. qPCR of *Lef-1* transcripts from the mesenchyme and epithelial tissues of E14.5 dissected tooth germs and isolated RNA, *N* = 3. **(F)** Incisor length measurements (mm) from the PN42 mice in **(A)**, *N* = 3. *p < 0.05, **p < 0.01.

*Lef-1* conditional knock-in (*Lef-1^*cKI*^*) mice were crossed with *Pitx2*^*Cre*^ mice to drive the over-expression of *Lef-1* in the dental and oral epithelium at E10.5. *Pitx2^*Cre*^/Lef-1^*cKI*^* or COEL of Lef-1 mice have a striking incisor over-growth phenotype, not due to malocclusions as the phenotype is observed in 100% of the mice (Figures 1A,B uCT images). The *COEL* mice have shorter nasal bone, snout length, frontal bone length, parietal bone length, cranial base length, cranial base angle, and ramus height (measurements not shown). However, the mandibular length is increased over WT mice. The *COEL* mandible is characterized by a reduced coronoid process and angular processes. There is also a general thinning or porosity in the region of the angular process (Figure 1B).

*Lef-1* expression remains in the tooth bud/oral epithelium during embryonic stages E14.5 in *COEL* embryos but is mostly mesenchymal in E14.5 WT embryos (Figure 1C). Lef-1 expression levels from IF sections were quantitated for fluorescence intensity (FI) and shown in Figure 1D (E14.5). *Lef-1* transcripts were quantitated in E14.5 dental mesenchyme and epithelium from WT and *COEL* embryos. As expected *Lef-1* transcripts increased in the epithelium of *COEL* embryos compared to WT (Figure 1E). Incisor length was measured and recorded in adult *COEL* mice compared to WT mice and show a large increase in overall length (Figure 1F). These data demonstrate ectopic expression of *Lef-1* in the oral epithelia after E14.5 correlating with dental and craniofacial growth defects.

**FIGURE 2:**
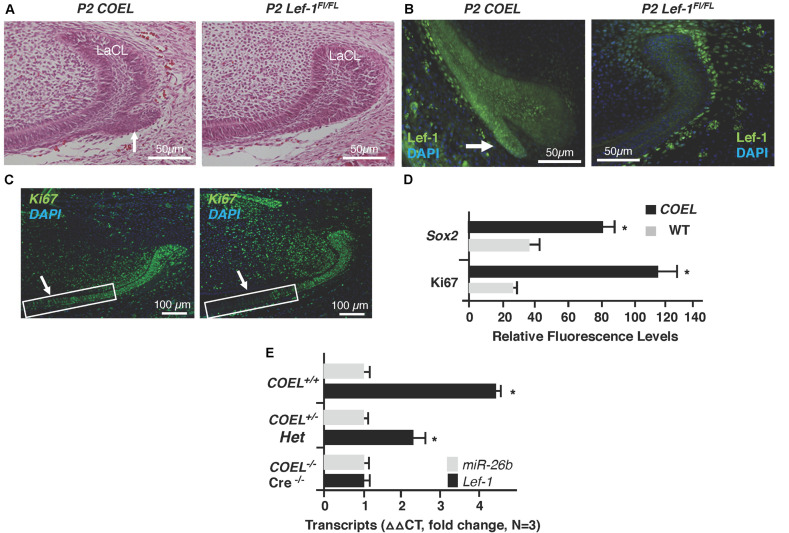
*Lef-1* over-expression creates an extra stem cell niche and increases cell proliferation in the lower incisor. **(A)** H&E staining of P2 sagittal sections of the mouse lower incisor showing the extra stem cell niche formed in the *COEL* mouse (white arrow). **(B)** Lef-1 is highly expressed in the new stem cell compartment of P2 *COEL* mice (white arrow). **(C)** Ki67 expression show by immunofluorescence was increased in the P2 *COEL* mice. **(D)** Sox2 and Ki67 positive cells were quantitated by ImageJ in *COEL* and WT mandibles. **(E)** *Lef-1* expression from P0 mandibles was increased in *COEL* mice with two copies of the transgene (*COEL*^+/+^) compared to one copy of *Lef-1* cDNA (*COEL*^±^) and WT or Cre negative mice, *N* = 3. *miR-26b* expression is not regulated by *Lef-1*. Labial cervical loop, LaCL. *p < 0.05.

In a previous report we documented a new stem cell compartment located in the LaCL of *COEL* mice (Figure 2A; Sun et al., 2016). This extra stem cell niche is created by the over-expression of *Lef-1* and contains high levels of Lef-1 expression (Figure 2B). Cell proliferation as measured by Ki67 staining was expanded in the P2 *COEL* transient amplifying cells and indicative of rapidly growing incisors (Figures 2C,D). Furthermore, Sox2 expression was significantly increased in the lower incisor LaCL (Figure 2D) (Sox2 staining shown in Sun et al., 2016). *Lef-1* transcripts in the mandible of *COEL* mice with one copy of the *Lef-1* cDNA transgene (*COEL*^±^) and two copies of *Lef-1* cDNA (*COEL*^+/+^) are increased proportionally (Figure 2E). Thus, ectopic *Lef-1* expression in the dental epithelium after E14.5 (when endogenous *Lef-1* expression transitions to the dental mesenchyme) results in an expanded stem cell niche and increased epithelial cell proliferation. Interestingly, the new stem cell compartment does not contain Ki67 positive cells.

**FIGURE 3:**
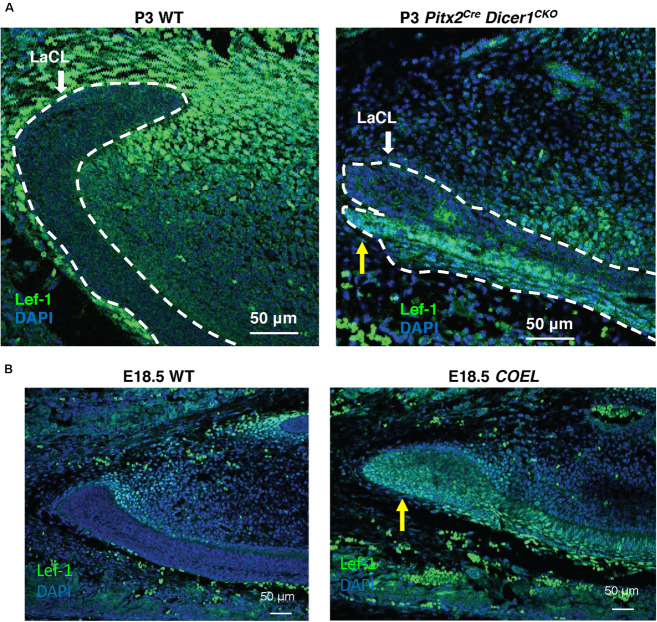
*Lef-1* expression was increased in the *Dicer1*^*cKO*^ incisor forming a new stem cell niche like *COEL* mice. **(A)** Lef-1 expression in sagittal sections of *Pitx2^*Cre*^/Dicer1^*cKO*^* P3 lower incisors. Low levels of Lef-1 expression in P3 WT epithelium were observed and increased Lef-1 expression was found in the branched stem cell niche of the P3 *Dicer1*^*cKO*^ mice (yellow arrow). **(B)** Lef-1 expression in the lower incisor of the E18.5 *COEL* lower incisor (yellow arrow). Labial cervical loop, LaCL.

We began studying the function of miRs during tooth development by conditionally knocking out *Dicer1* using the *Pitx2*^*Cre*^ and found these mice had multiple enamel-free incisors (Cao et al., 2010). The complete ablation of mature miRs in these mice resulted in branched incisors caused by the formation of extra stem cell niches (Cao et al., 2010), similar to the *COEL* mice. We hypothesized that this might result from ectopic *Lef-1* expression in the mutant LaCL and thus assayed for Lef-1 expression in P3 *Pitx2^*Cre*/^Dicer1^*CKO*^* mice. Indeed, like in the *COEL* mice, Lef-1 expression was associated with the formation of an additional stem cell niche (Figure 3A, yellow arrow). This extra niche was used to create a branched incisor (Cao et al., 2010). The E18.5 *COEL* embryos also show increased Lef-1 expression in a similar region of the LaCL producing a new stem cell niche compartment (Figure 3B, yellow arrow). These results show that miRs are controlling the organization of the stem cell niche by regulating *Lef-1* expression.

### The *miR-26b* Over-Expression Mice Have Craniofacial Defects That Phenocopy *Lef-1* Knockout Mice

We have shown that *miR-26b* directly targets *Lef-1* and represses colon cancer cell proliferation (Zhang et al., 2014). Furthermore, *miR-26b* represses the expression of the *Lef-1* target genes *cyclin D1* and *cMyc* (Zhang et al., 2014). To determine miR expression profiles, we analyzed the dental mesenchyme and epithelial tissues from P0 and E14.5 mice and embryos, respectively. We identified several miRs that were differentially expressed in the epithelial tissue compared to mesenchyme (Cao et al., 2010). *miR-26b* was not highly expressed prior to E14.5 in the dental epithelium at a time when *Lef-1* expression was transitioning to the mesenchyme. However, *miR-26b* expression was increased after E14.5 in the dental epithelium, which may act to decrease *Lef-1* expression at this stage (Cao et al., 2010).

**FIGURE 4:**
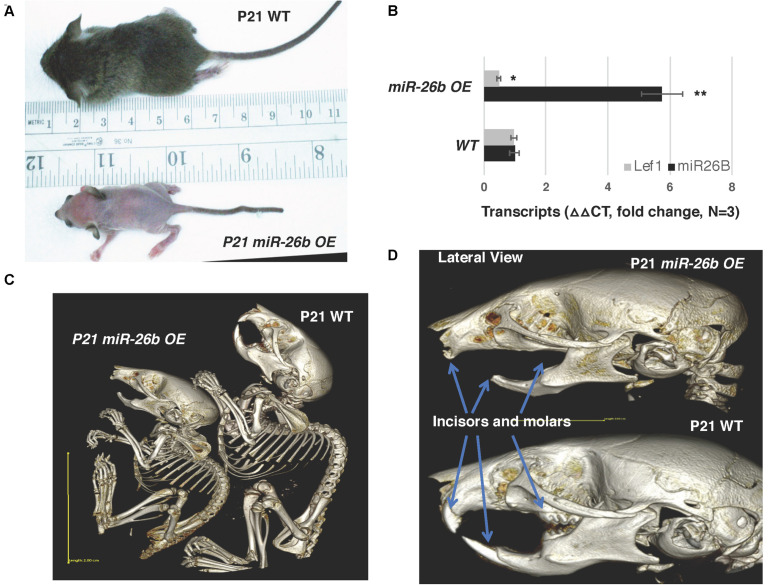
*miR-26b OE* mice have defects in ectodermal organ development. **(A)** *miR-26b* over-expression (OE) mice are small, have a loss of hair and a crooked tail like *Lef-1* knockout mice. **(B)** *miR-26b* is over-expressed in the *miR-26b OE* mouse mandible with a decrease in *Lef-1* transcripts shown by qPCR. **(C)** μCT whole body images of the P21 *miR-26b OE* and WT mice. **(D)** μCT head images showing a lack of incisors and molars and bone defects in the P21 *miR-26b* mice compared to WT. *p < 0.05; **p < 0.01.

The *miR-26b* transgenic over-expression (OE) mouse has an incomplete penetrance. *miR-26b^*Tg*±^* (Het) mice are mostly normal, but under-represented in live births and late embryonic stages, and most of the double transgenic *miR-26b^*Tg+/Tg+*^* mice die early in embryonic development, before E14.5. However, some mice are born and live to weaning date, but then cannot process solid food. These mice are small, with a lack of fur, and a very defined crook in the tail (Figure 4A). Interestingly, these mice resemble the *Lef-1* general knockout mice (Van Genderen et al., 1994). *miR-26b* expression is increased and endogenous *Lef-1* transcripts are decreased in the *miR-26b OE* mice (Figure 4B).

These mice lack teeth, including molars and incisors, shown by uCT imaging (Figures 4C,D). Full body uCT images reveal skeletal and bone defects in P21 mice (Figures 4C,D). The cranial base and cranial breath measurements are essentially identical in the *miR-26b OE* mice. However, the *miR-26b OE* mice have a shorter nasal bone, snout, frontal bone, parietal bone, cranial breath, and cranial base length compared to WT mice (measurements not shown). The cranial base angle and ramus height are also decreased in the *miR-26b OE* mice compared to WT mice. In contrast to the *COEL* mice, the *miR-26B OE* mice have a decreased mandibular length compared to WT mice (measurements not shown).

### *miR-26b OE* Inhibits *Lef-1* Expression and Arrests Tooth Development

**FIGURE 5:**
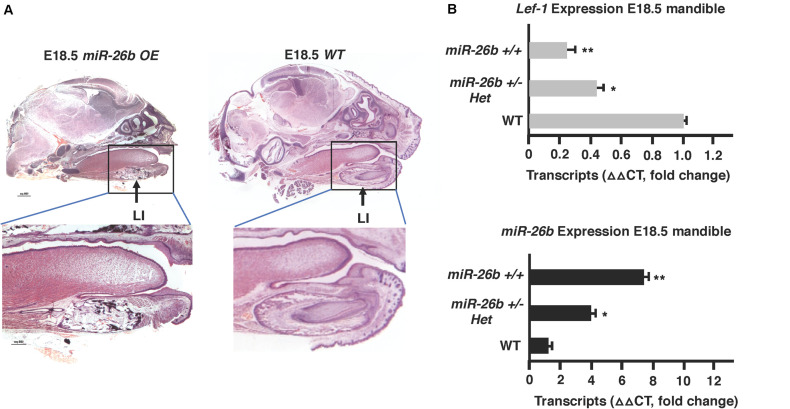
Tooth development is arrested in the *miR-26b OE* embryo. **(A)** Sagittal sections and H&E staining of the E18.5 *miR-26b OE* and WT heads. The boxed region is magnified to show a lack of a lower incisor tooth germ formation. **(B)** *miR-26b* expression correlates with decreased *Lef-1* expression in the mandibles of the mice in **(A)** by qPCR (*N* = 3, *p < 0.05, **p < 0.01).

The μCT images show that the *miR-26b OE* P21 mice do not have incisors or molars. *miR-26b OE* embryos were harvested, sectioned and H&E stained to examine tooth development at an earlier stage. The lower and upper incisors and molar tooth germs were completely absent at E18.5 (Figure 5A). We show a dose response for *miR-26b* expression and *Lef-1* expression. In mice with one copy of the *miR-26b* transgene, *Lef-1* expression is decreased approximately 50% (Figure 5B). In mice with two copies of *miR-26b* we show that *Lef-1* expression is further decreased and the E18.5 *miR-26b OE* embryos shown without tooth germs have two copies of the *miR-26b* transgene (Figures 5A,B). These data demonstrate that *miR-26b* is a potent regulator of tooth and cranial bone development.

### The Wnt Signaling Effectors and Pathway Are Regulated by *miR-26b*

**FIGURE 6:**
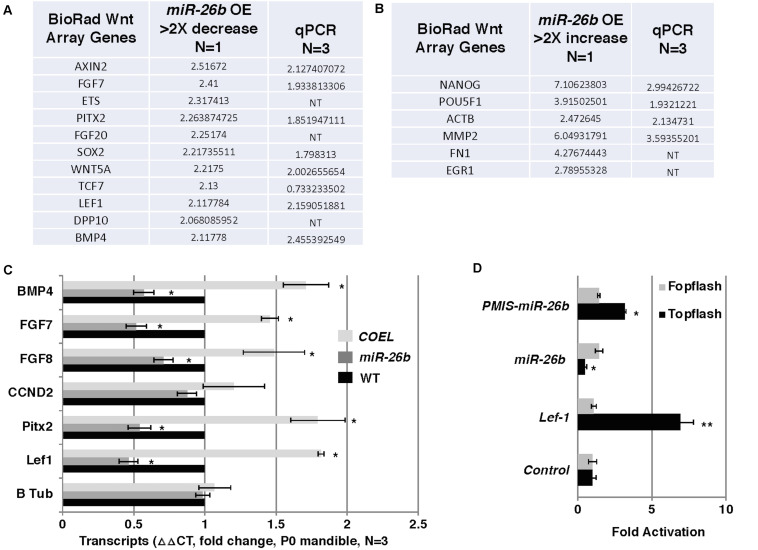
Wnt responsive genes are regulated by *miR-26b*. **(A)** Wnt PCR array was used to probe for Wnt genes regulated by *miR-26b*. Specific genes are shown that were decreased in the *miR-26b OE* mouse, *N* = 1. The genes were validated by qPCR (*N* = 3). **(B)** Wnt array genes that were increased in *miR-26b OE* mice (*N* = 1) and validated by qPCR. **(C)** Selected genes were assayed by qPCR from RNA isolated from P0 mandibles of *COEL*, *miR-26b OE*, and WT mice, *N* = 3. **(D)** *Lef-1* cDNA, *miR-26b*, and *PMIS-miR-26b* plasmid DNAs were transfected into HEK 293 cells with either Topflash or Fopflash luciferase reporter constructs. Luciferase activity was recorded and expressed as fold activation compared to controls without *Lef-1*, *miR-26b*, or *PMIS-miR-26b* expression (*N* = 3, *p < 0.05, **p < 0.01).

Lef-1 is known to play a role in Wnt signaling and transcriptional activation. We tested for the possibility that reductions in *Lef-1* expression levels would cause alterations in Wnt signaling. Wnt signaling in the *miR-26b OE* mouse was analyzed by isolating RNA and using a Bio-Rad Wnt signaling array. There were reductions in 10 of the 80 Wnt target genes, including *Fgf7/8* and *Bmp4* and an increase in several genes involved in stemness, such as *Nanog* and *Pou5F1* (Figures 6A,B). These results were verified by qPCR from P0 mandible tissue. The *COEL* mouse showed upregulation of *Fgf7/8* and *Bmp4* by qPCR (Figure 6C). Lef-1 regulates several key Wnt targets and Wnt signaling genes are upregulated by OE of *Lef-1* and reduced by OE of *miR-26b* (Figure 6C). We further demonstrate the effect of *Lef-1* and *miR-26b* transcriptional regulation using the Topflash luciferase reporter (Zhang et al., 2011). A *Lef-1* cDNA, a *miR-26b* construct and an inhibitor of endogenous *miR-26b* (*PMIS-miR-26b*) were transfected into HEK293 cells with the Topflash or Fopflash control reporter constructs. As expected Lef-1 activated the Topflash reporter but did not activate the Fopflash control reporter (Figure 6D). *miR-26b* expression inhibited the Topflash reporter, which contains 7 *Lef-1* binding elements, but has no effect on the control Fopflash reporter (Figure 6D). When endogenous *miR-26b* was inhibited using the *PMIS-miR-26b* construct the Topflash reporter was activated due to increased endogenous *Lef-1* activity (Figure 6D). Together these data show that *miR-26b* is regulating the Wnt signaling pathway through the regulation of *Lef-1* expression.

### *miR-26b OE* Rescues the *COEL* Phenotype

**FIGURE 7:**
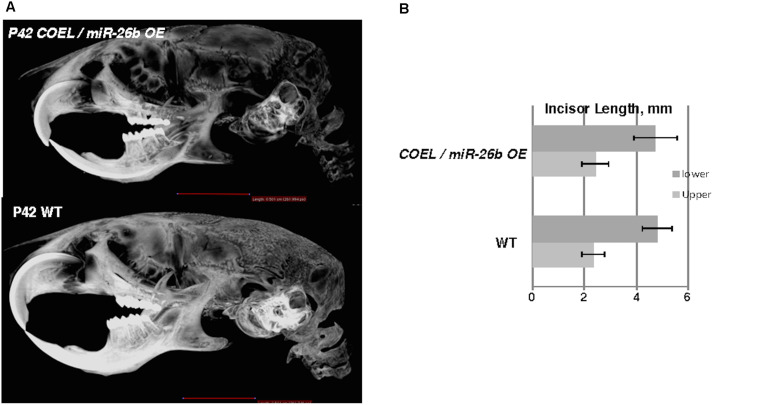
*miR-26b OE* rescues the *COEL* phenotype. **(A)** μCT head images of PN42 *COEL/miR-26b OE* rescue mice and WT mice. **(B)** Incisor length measurements demonstrate that the length of rescue mice incisors are similar in length to WT mice, *N* = 3.

The over-expression of *miR-26b* phenocopied *Lef-1^–/–^* mice and regulated *Lef-1* expression *in vivo.* We hypothesized that crossing the *miR-26b OE* mice with *COEL* mice might result in a rescue of the tooth phenotype. Indeed, these mice (*COEL/miR-26b OE*) resulted in 100% penetrance of the rescued phenotype as all mice had normal tooth development. The μCT images show the restoration of the incisors and molars (Figure 7A). The measurements of the P42 *COEL/miR-26b OE* lower incisor length were similar as observed in P42 WT mice (Figure 7B). Overall growth defects were not corrected (data not shown) as the mice continue to be smaller than wild-type littermates. Also, OE of *Lef-1* corrected the lethality and tooth defects in the *miR-26b OE* mouse strain. While liter sizes were small, we obtained Mendelian frequencies of all genotypes when crossing the *COEL* and *miR-26b OE* strains and no mice were born with defects in incisors or molar growth.

**FIGURE 8:**
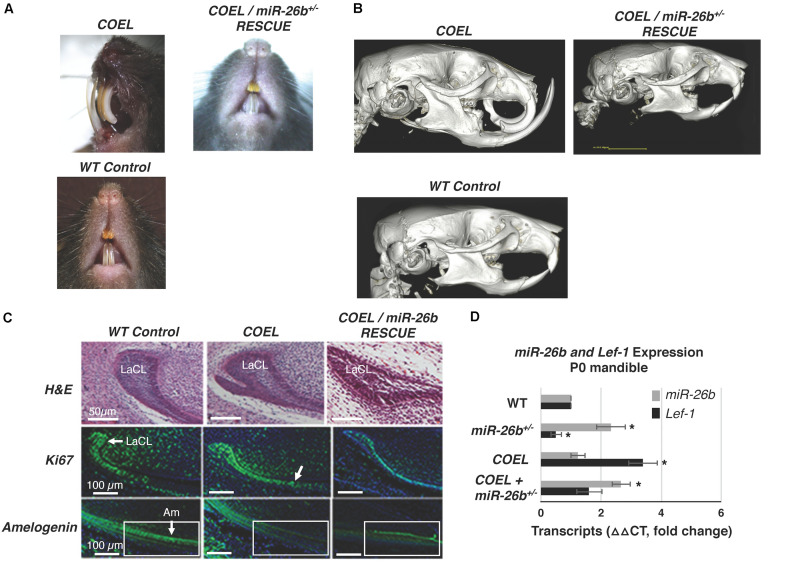
Comparison of the *COEL*, *COEL/miR-26b* rescue and WT mice demonstrating normal incisor development in the rescued mice. **(A)** Mice heads with incisor phenotypes are shown, **(B)** μCT head images comparing the *COEL*, *COEL/miR-26b* rescue, and *Cre* negative control mice bone and tooth development. **(C)** Sagittal sections of P2 lower incisors of the three mice showing the *COEL* extra stem cell compartment and rescue by *miR-26b*, H&E staining; Ki67 staining shows that cell proliferation in the rescue mice is similar to WT mice; and Amelogenin expression is similar in the rescue mice as observed in WT mice. The boxed region is shown for comparison. **(D)** P0 mandibles were processed for RNA and analyzed for *Lef-1* and *miR-26b* expression from WT, *COEL*, and *COEL miR-26b*^±^ mice. (*N* = 3, *p < 0.05). Labial cervical loop, LaCL; Ameloblasts, Am.

We show representative P42 mice with *COEL*, control and *COEL/miR-26b OE* rescue mice phenotypes (Figure 8A). For comparison, separate μCT images of these phenotypes are shown to contrast the tooth and cranial bone phenotypes (Figure 8B). We sectioned P0 mice to determine if *miR-26b* OE could correct the defects of the LaCL and the developmental defects in the *COEL* mouse. The LaCL morphology was mostly restored to the WT structure in the *COEL/miR-26b OE* mouse (Figure 8C). The altered Ki67 and *Amelogenin* expression shown in the *COEL* mice were corrected in the rescue mice and similar to the wild-type control (Figure 8C). Furthermore, *Lef-1* expression in the rescue mice was similar to WT mice (Figure 8D). These data demonstrate a genetic association between the *COEL* and *miR-26b OE* murine phenotypes. Furthermore, we show that *miR-26b* specifically regulates *Lef-1* and that *Lef-1* is a major target of *miR-26b* during craniofacial and tooth development. Specifically, we have identified a *Lef-1* dosage effect required for normal tooth development and craniofacial development modulated by *miR-26b*.

## Discussion

Stem cells derived from stem cell niches contribute to the regeneration of mature tissue types in many different organs, including the trachea, lungs and teeth, amongst others (Harada et al., 1999; Tummers and Thesleff, 2003; Chistiakov, 2010; Juuri et al., 2012; Sun et al., 2016). These niches are formed in developing embryos, and must be maintained throughout life by symmetric cellular divisions that produce daughter pluripotent stem cells (Morrison, 2008). Another equally important behavior of the cells in a stem cell niche is the production of differentiated daughter cells by asymmetric cell division, which then take the place of damaged cells in regenerative organs, in order to allow the organ to continue to function (Knoblich, 2008). How these behaviors are regulated is an important question for stem cell biologists, who seek to ultimately apply stem cell therapies to a host of diseases in which the ability of the body to sustain the production of cell types required for normal function becomes impaired. Currently, much is known about the transcriptional programs that maintain a stem like state (Young, 2011). However, more work is required to investigate how cells can turn off these stem cell programs in order to differentiate. Historically, transcription factors have been thought of as the key determinants of cell state (Iwafuchi-Doi and Zaret, 2016). Recently, however, miRs have become appreciated as playing a role in stem cell differentiation (Schwamborn, 2009). The ability to co-opt miR expression to reprogram and control the differentiation of naive cells into different cell types is an important tool required to create artificial organs and repair diseased tissues, saving millions of lives and public dollars.

Several miR families have been implicated in tooth development by our group and others. *miR-26b* is of interest because it is highly expressed during specific stages of tooth development. We found its expression was absent from the oral epithelium and DESCs of the LaCL in the lower murine incisor prior to E14.5, when *Lef-1* is expressed. However, *miR-26b* is highly expressed at later stages in all dental epithelial tissues, including the LaCL indicating that it may play a functional role in DESC differentiation. The *COEL* mouse was generated to understand the function of tissue specific *Lef-1* expression and demonstrates that *Lef-1* regulates DESC proliferation. While Lef-1 is known to control stem cell self-renewal and stem cell compartments in the epidermis and hair follicles, we show it is also required for the formation of a DESC niche. Interestingly, the *miR-26b* OE mouse completely inhibits tooth development by targeting *Lef-1* expression, demonstrating a critical role for *miR-26b* during embryonic development. These are two new mouse models that will allow us to understand the genetic and functional activities of both *Lef-1* and *miR-26b*.

### *Lef-1* in Tooth Development

Previous studies have shown that *Lef-1* is regulated by FGF signaling and is required for early tooth development (Kratochwil et al., 1996, 2002; Sasaki et al., 2005). *Lef-1* deficiency results in arrested tooth morphogenesis at the late bud stage (Van Genderen et al., 1994), and that *Lef-1* is required only transiently in the dental epithelium for tooth development (Kratochwil et al., 1996). *Lef-1* expression is shifted to mesenchymal cells/tissues surrounding the epithelium at the bud stage, although low levels of *Lef-1* expression remain in the dental epithelium (Kratochwil et al., 1996; Sasaki et al., 2005). A developmental mechanism for this transition was recently shown using the *COEL* mice thus, Lef-1 expression after E14.5 in the dental epithelium creates a new LaCL stem cell niche and abnormal “tusk-like” incisors (Sun et al., 2016). Furthermore, over-expression of *Lef-1* partially rescued tooth arrest in *Sox2*^*cKO*^ embryos (Sun et al., 2016). Both *Sox2* and *Lef-1* are markers of early craniofacial development and are expressed in the oral and dental epithelium (Sasaki et al., 2005; Juuri et al., 2012, 2013; Zhang et al., 2012; Sun et al., 2016). These data demonstrated that *Lef-1* can partially replace *Sox2* as a potential stem cell factor to both initiate and maintain the lower incisor LaCL stem cell niche.

In this report, we show that the new LaCL stem cell compartment highly expresses *Lef-1* and that this new compartment does not contain actively dividing cells, whereas cell proliferation is increased in the transient amplifying cells. The new stem cell compartment contains quiescence cells that are partitioned adjacent to the LaCL and these cells may provide progeny to the outer enamel epithelium and the stratum intermedium cell layers as well as the inner enamel epithelium. Interestingly, the new stem cell compartment does not form until birth or shortly before, suggesting that continued *Lef-1* expression in the dental epithelium contributes to stem cell development but not maintenance. The rapid growth of the *COEL* incisors may indicate that the new stem cell niche provides an additional progenitor cell source that contributes to increased cell proliferation in both the outer and inner enamel epithelial cell layers. We are currently exploring *Lef-1*-mediated cell reprogramming and tissue/tooth regeneration as Lef-1 may act as a master transcription factor for tooth development.

These data provide a molecular mechanism for why *Lef-1* expression is required for early formation of the LaCL epithelial stem cell niche. In normal incisor development *Lef-1* expression is decreased in the dental epithelium at E14.5 and *Lef-1* expression transitions to the dental mesenchyme. It is well established that the odontogenic potential shifts from the dental epithelium after E12.5 to the mesenchyme during early murine tooth development (Zhang et al., 2005). Interestingly, COEL of *Lef-1* in the mesenchyme also results in an incisor overgrowth phenotype (unpublished data). *Lef-1* may be playing an essential role in this transition. As *Lef-1* expression increases in the dental mesenchyme this correlates with the odontogenic potential shift to the mesenchyme. Furthermore, we have shown that *Lef-1* over-expression in the LaCL results in an increase in *Sox2* expression in the new stem cell compartment (Sun et al., 2016). Therefore, *Lef-1* may act as an initial stem cell factor during ectodermal organ development to set up specific cell fates and regulate gene expression required for the maintenance and compartmentalization of the dental stem cell niche.

### The Role of MicroRNAs in Tooth Development and Regulation of *Lef-1* Expression

The conditional inactivation of the microRNA processing gene, *Dicer1*, resulted in tooth defects including, extra teeth, branched teeth, abnormal tooth shape, and loss of enamel due to impaired ameloblast differentiation (Cao et al., 2010; Michon et al., 2010). However, ablating all mature miRs from the dental epithelium offered clues as to the actions of miRs but did not identify which specific miRs were involved in the early process of tooth development. We isolated dental epithelial tissues and profiled them for miR expression and found that *miR-26b* was also differentially expressed in the dental epithelium (Cao et al., 2010). Prior to E14.5 *miR-26b* is not expressed in the LaCL stem cell niche, however after E14.5 *miR-26b* expression is increased and its expression in the epithelium remains until birth. *miR-26b* expression is inversely correlated with *Lef-1* expression in the developing tooth.

The role of *miR-26b* during development was unknown until this report, as no mouse models were generated and more importantly nothing is known about the role of *miR-26b* in dental stem cell proliferation and tooth morphogenesis. Murine *miR-26b* is expressed on chromosome 1 and is an intragenic miR and has been reported to be involved in multiple cancers. We have previously shown that *miR-26b* directly targets *Lef-1* and regulates cell proliferation through the *Lef-1* target genes *Cyclin D1* and *cMyc* (Zhang et al., 2014). When we checked our *Pitx2^*Cre*^/Dicer1* conditional knockout mice for *Lef-1* expression, we found increased *Lef-1* expression (Cao et al., 2010) and it was also associated with the extra stem cell compartment we identified in the *Dicer1^*cKO*^* embryos.

### *miR-26b* Over-Expression Inhibits *Lef-1* Expression and Results in Ectodermal Organ Defects

**FIGURE 9:**
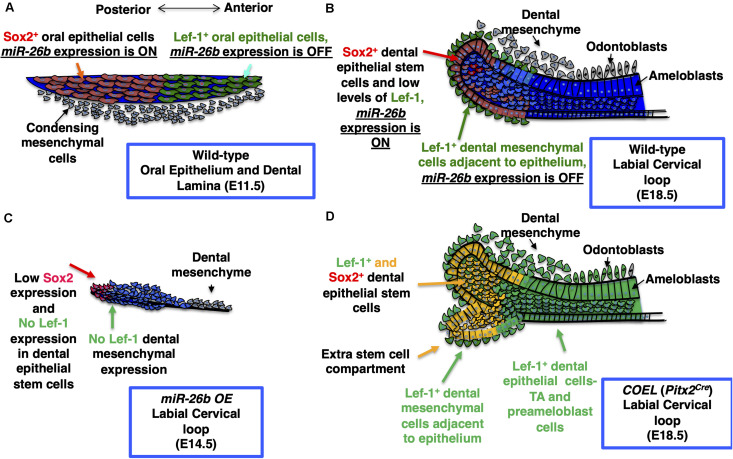
Model for the role of *miR-26b* and *Lef-1* in the lower incisor during development. **(A)** The normal expression of *Sox2* and *Lef-1* during E11.5 dental placode development. **(B)** The normal lower incisor development at E18.5 showing the WT labial cervical loop (LaCL), *Sox2* and *Lef-1* expression and *miR-26b* expression domains. **(C)** *miR-26b OE* inhibits tooth bud development with no Lef-1 and reduced Sox2 expression at E14.5 in the LaCL. **(D)** An extra stem cell compartment is formed in the E18.5 *COEL* LaCL expressing *Sox2* and *Lef-1* and the dental epithelial cells express *Lef-1* causing an increase in dental epithelial cell proliferation and increased incisor growth. TA refers to the Transient Amplifying Cells.

Because *miR-26b* targets *Lef-1* expression and Lef-1 is a critical factor for early tooth development we asked if *miR-26b* over-expression affected tooth development. The ectopic expression of *miR-26b* occurs early in development using a *EF1a* promoter to over-express *miR-26b* in all tissues. We show that *miR-26b OE* mice have arrested tooth development. Therefore, during tooth development the epithelial expression of *Lef-1* is inhibited prior to its expression in the mesenchyme by ectopic expression of *miR-26b*. The effect of over-expressing *miR-26b* demonstrates its role during embryonic development is to regulate *Lef-1*, as most of the mouse defects are associated with tissues that require *Lef-1* for normal development. Therefore, *miR-26b* only regulates specific tissues and the genes required for *Lef-1*-dependent developmental processes. The *miR-26b OE* mice have a loss of both molars and incisors as well as a loss of hair and other defects associated with *Lef-1* deletion in mice. Interestingly, other defects such as cranial bone defects or decreased growth of bones could be affected by mesenchymal *miR-26b* expression or by affecting pituitary development. We have shown that *miR-26b* targets *Lef-1*, which modulates Pituitary Transcription Factor 1 (Pit-1) expression (Zhang et al., 2010). A similar mechanism occurs in the pituitary where *miR-26b* regulates *Pit-1* expression by inhibiting *Lef-1* expression to promote *Pit-1* lineage differentiation during pituitary development. Curiously, in the *Pitx2^*Cre*^/Dicer1^*cKO*^* mutant pituitary we also reported an abnormal branching phenotype of the pituitary with a loss of mature miRs (Zhang et al., 2010). We postulated that *miR-26b* was targeting the Wnt pathway for pituitary development. In this report, we show that *miR-26b OE* in the mandible reduced the expression of multiple Wnt target genes and upregulated several stemness genes. Thus, in normal development *miR-26b* inhibits *Lef-1* expression to allow for cell differentiation and inhibit proliferation. These data demonstrate a unique role for *miR-26b* in ectodermal organ development by regulating *Lef-1* expression in a temporal and spatial manner to ensure stem cell niche maintenance and cell differentiation. We propose a model for *Lef-1* and *miR-26b* function during incisor development (Figure 9).

### *miR-26b* Over-Expression Rescues the *COEL* Phenotype

A previous report demonstrated that exogenous Fgfs were able to rescue the phenotype of *Lef-1* mutant teeth (Kratochwil et al., 2002). We demonstrate that *miR-26b OE* rescues the *COEL* phenotype and results in normal tooth development. Because our *COEL* mouse model uses a *Lef-1* cDNA construct it is not regulated by *miR-26b* however, endogenous *Lef-1* expression can be inhibited allowing us to differentially regulate *Lef-1* expression and rescue *Lef-1 OE* phenotypes by inhibiting endogenous *Lef-1*. These unique transgenic mouse models demonstrate an effective *Lef-1* dosage requirement for ectodermal organ development. Thus, *miR-26b* over-expression only targets endogenous *Lef-1* expression, because it targets the 3′UTR of *Lef-1*. Whereas the *COEL* mouse conditionally expresses a cDNA lacking the *Lef-1* 3′UTR and is not regulated by *miR-26b*. Therefore, the rescue is due to normal levels of *Lef-1* cDNA expression, because *miR-26b* inhibits the endogenous *Lef-1* expression in the *COEL/miR-26b* mouse. These experiments show that *Lef-1* expression is tightly regulated for normal ectodermal development. It is interesting that the rescue mice have an overall growth phenotype suggesting that not all defects are affected by this rescue. More research is required to understand the role of *miR-26b* and *Lef-1* during development of multiple organs and tissues.

## Data Availability Statement

The raw data supporting the conclusions of this article will be made available by the authors, without undue reservation.

## Ethics Statement

The animal study was reviewed and approved by Institutional Animal Care and Use Committee (IACUC). Mice were housed and experiments performed according to the Office of Animal Resources guidelines at the University of Iowa.

## Author Contributions

SE performed the experiments, analyzed the data, prepared the manuscript, and contributed to the design of the study. TS and YS performed the experiments and analyzed the data. MS collected and analyzed the data. BA contributed to the design of the study, data interpretation, and acquisition, and prepared the manuscript. All authors contributed to the article and approved the submitted version.

## Conflict of Interest

The authors declare that the research was conducted in the absence of any commercial or financial relationships that could be construed as a potential conflict of interest.
